# A pilot study to determine whether combinations of objectively measured activity parameters can be used to differentiate between mixed states, mania, and bipolar depression

**DOI:** 10.1186/s40345-017-0076-6

**Published:** 2017-03-01

**Authors:** Jan Scott, Arne E. Vaaler, Ole Bernt Fasmer, Gunnar Morken, Karoline Krane-Gartiser

**Affiliations:** 1 Academic Psychiatry, Institute of Neuroscience, Newcastle University, Newcastle upon Tyne, UK; 2 Department of Neuroscience, NTNU, Norwegian University of Science and Technology, Trondheim, Norway; 3 Department of Psychiatry, St. Olav’s University Hospital, Trondheim, Norway; 4 Department of Clinical Medicine, Section for Psychiatry, Faculty of Medicine and Dentistry, University of Bergen, Bergen, Norway; 5 Division of Psychiatry, Haukeland University Hospital, Bergen, Norway

**Keywords:** Actigraphy, Non-linear dynamics, Mixed states, Discriminant analysis, Classification, Illness phase

## Abstract

**Background:**

Until recently, actigraphy studies in bipolar disorders focused on sleep rather than daytime activity in mania or depression, and have failed to analyse mixed episodes separately. Furthermore, even those studies that assessed activity parameters reported only mean levels rather than complexity or predictability of activity. We identified cases presenting in one of three acute phases of bipolar disorder and examined whether the application of non-linear dynamic models to the description of objectively measured activity can be used to predict case classification.

**Methods:**

The sample comprised 34 adults who were hospitalized with an acute episode of mania (*n* = 16), bipolar depression (*n* = 12), or a mixed state (*n* = 6), who agreed to wear an actiwatch for a continuous period of 24 h. Mean level, variability, regularity, entropy, and predictability of activity were recorded for a defined 64-min active morning and active evening period. Discriminant function analysis was used to determine the combination of variables that best classified cases based on phase of illness.

**Results:**

The model identified two discriminant functions: the first was statistically significant and correlated with intra-individual fluctuation in activity and regularity of activity (sample entropy) in the active morning period; the second correlated with several measures of activity from the evening period (e.g. Fourier analysis, autocorrelation, sample entropy). A classification table generated from both functions correctly classified 79% of all cases based on phase of illness (*χ* ^2^ = 36.21; *df* 4; *p* = 0.001). However, 42% of bipolar depression cases were misclassified as being in manic phase.

**Conclusions:**

The findings should be treated with caution as this was a small-scale pilot study and we did not control for prescribed treatments, medication adherence, etc. However, the insights gained should encourage more widespread adoption of statistical approaches to the classification of cases alongside the application of more sophisticated modelling of activity patterns. The difficulty of accurately classifying cases of bipolar depression requires further research, as it is unclear whether the lower prediction rate reflects weaknesses in a model based only on actigraphy data, or if it reflects clinical reality i.e. the possibility that there may be more than one subtype of bipolar depression.

**Electronic supplementary material:**

The online version of this article (doi:10.1186/s40345-017-0076-6) contains supplementary material, which is available to authorized users.

## Background

The new edition of the Diagnostic and Statistical Manual (DSM-5) identifies increased activity and energy alongside mood change as cardinal symptoms of (hypo)mania (American Psychiatric Association 2013). Actigraphy has well-established validity in the objective measurement of motor activity (Ancoli-Israel et al. 2015), however, its use in research in bipolar disorders (BD) has primarily focused on the analysis of sleep patterns [for reviews see: Geoffroy et al. (2015); Ng et al. (2015); De Crescenzo et al. (2016); Scott et al. (2016a)]. When actigraphy is used to examine sleep-wake cycles or 24-h rest-activity patterns in community or outpatient samples, differences emerge not only between BD cases and comparator groups [including healthy controls or unipolar depression (UP), etc.], but also between BD cases and their non-BD relatives, between depression cases with or without a family history of BD, and between euthymic, depressive, and mania phases of BD (Harvey et al. 2005; Jones et al. 2005; St-Amand et al. 2013; Salvatore et al. 2008; Indic et al. 2011; Scott 2011; Faurholt-Jepsen et al. 2012; Robillard et al. 2013; Gonzalez et al. 2014; Janney et al. 2014; McGlinchey et al. 2014; McKenna et al. 2014; Merikangas et al. 2014; Pagani et al. 2016; Gershon et al. 2016; Grierson et al. 2016; Scott et al. 2016b). Most, but not all, outpatient and inpatient studies of actigraphy suggest that, compared to euthymia or inter-episode states, BDep is more likely to be characterized by lower mean levels of activity, more variability, or a delay in the sleep-wake cycle; in contrast, mania typically shows reduced rhythmicity or regularity (such as reduced difference in the activity levels during the day versus the night) and less predictable patterns of activity (Kupfer et al. 1974; Weiss et al. 1974; Wehr et al. 1980; Salvatore et al. 2008; Robillard et al. 2013; Gonzalez et al. 2014; Krane-Gartiser et al. 2014; Gershon et al. 2016). However, findings are inconsistent and, in a recent systematic review, Scott et al. (2016a) noted that it was difficult to evaluate patterns of activity across all phases of BD for four main reasons. First, there is virtually no actigraphic data available on activity patterns in mixed states. Indeed, the only study to include these cases to date (Salvatore et al. 2014), did not specify the number of cases meeting the criteria for a mixed state, and did not analyse data on mixed states separately from mania. Second, few objective studies of BD report measures of activity beyond the mean level over 24 h, and this was especially true for BDep where constructs that reflect ‘non-uniform’ patterns of activity are under-explored (Gershon et al. 2016). Third, the use of non-linear mathematical models to explore the regularity, predictability, or complexity of activity patterns is limited (Salvatore et al. 2008; Indic et al. 2011; Gonzalez et al. 2014; Merikangas et al. 2014; Pagani et al. 2016; Gershon et al. 2016; Grierson et al. 2016; Scott et al. 2016b) and only Krane-Gartiser et al. (2014, 2015, 2016) have used such approaches with inpatient samples that comprised cases with mood disorders only. Fourth, we lack insights into whether specific combination of activity measures may classify different phases of BD and only one study to date (of paediatric BD vs. attention deficit hyperactivity disorder) has attempted to explore whether combinations of rest-activity variables can be used to discriminate between diagnostic groups (Faedda et al. 2016).

The relatively slow uptake of non-linear mathematical models in BD research is disappointing, as they are well-established in general medicine and have been critical to the development of a more sophisticated understanding of the nature of system dynamics than can be achieved by traditional approaches alone (Goncalves et al. 2015). For example, in cardiology, these approaches have enabled clinicians and researchers to build a more complete picture of the nature of heart rate variability and its consequences. The limited use of statistical approaches to classification is more understandable, as most clinical studies of actigraphy in BD are based on modest samples, which can limit the utility of techniques such as machine learning (Orru et al. 2012). However, other approaches have been used to subclassify cases in psychiatry in relatively small samples. For example, discriminant function analysis (DFA) has been applied to determine whether neurocognitive profiles can be used to categorize cases such as schizophrenia, schizoaffective disorder, or BD (van Rheenen et al. 2016), and to identify which components of a multi-assay biological test could be used in a screening procedure for mood disorders (Yamamori et al. 2016).

The current pilot study represents a first attempt to begin to address the identified gaps in our knowledge about activity patterns across BDep, mania, and mixed episodes. We extracted data on actigraphy parameters that reflected mean levels, variability, regularity, and predictability of activity at different times of day (morning and evening) and used DFA to explore if any combinations of these activity measures can be used to classify BD cases based on phase of illness. We emphasize that the goal of this paper was to explore the viability of using this approach to classify alongside non-linear models as, to our knowledge, this is the first time DFA has been used for actigraphy measures. This ‘proof of principle’ study represents an important initial step in determining whether the selected measures of activity and the analytic strategy proposed could be applied to larger datasets (obtained from a single large-scale study or pooled data from several independent studies).

## Methods

The study procedures have been described in detail elsewhere (Krane-Gartiser et al. 2014, 2015), so we briefly summarize the key elements.

### Sample

With ethical approval, adults with BD who were admitted as psychiatric inpatients to St. Olav’s University Hospital (in Trondheim, Norway), and who were willing and able to give written informed consent to participate in research, were invited to take part in an assessment of sleep-wake activity patterns recorded consecutively for 24 h during the earliest stage of their admission (usually between day one and three following hospitalization).

Thirty-four patients aged >18 years had (1) actigraphy data available on the six selected activity parameters and (2) a BD diagnosis that met ICD-10 ‘Criteria for research’: 16 were manic (F31.1–F31.2), 12 had BDep (F31.3–F31.5), and six had a mixed episode (F 31.6).

### Actigraphy

#### Recordings of motor activity

All recordings began during daytime hours (mean 12:41 PM; SD 2.55 h), and activity counts were recorded for one minute intervals using a wrist-worn actigraph (Actiwatch, Philips Respironics, Murrysville, USA). The average duration of continuous actigraphic recording was 22 h.

For each case, we selected actigraphy data recorded between 6 a.m. to midnight and separated this period into morning and evening epochs. Morning epochs were defined to occur between 6 a.m. and 3 p.m., and evening epochs between 3 p.m. and midnight. Next, we searched each recording for periods of continuous motor activity in the morning and evening of 64-min duration. This timeframe was chosen to comply with the Fourier analysis (see later), which requires sequence lengths to be potencies of two (32, 64, 128, etc.). The active morning period was searched from the start of the series, and the active evening period from the end of the series. For each participant, we selected the first period of 64 min not containing more than two consecutive minutes of zero activity counts (if there was no such period, we searched for sequences with no more than 3 consecutive minutes with zero activity, etc.) (Hauge et al. 2011).

#### Activity parameters

From the activity counts (measured as counts per minute) in the actigraph software programme (Actiware, version 5.70.1), we calculated the following parameters for the 64-min continuous active morning and evening periods:

- mean activity count per minute.
- intra-individual fluctuations in activity measured using the standard deviation (SD) for each time series.
- variability in the time series using the root mean squares of successive differences (RMSSD), which describes the difference in successive activity counts from minute to minute.
- Autocorrelation (at lag 1), which is a mathematical tool that helps to expose repeating patterns (e.g. rain today may predict it will rain tomorrow, etc.). An autocorrelation function refers to the correlation of a time series with its own past and future values. The autocorrelation at lag 1 is the correlation of this time series with itself lagged minute to minute. Values closer to one indicate a stronger correlation.
- Sample entropy was used as a non-linear measure of the degree of regularity (complexity) of a time series and was estimated using software that is available online (Physio Toolkit; http://www.physionet.org). This measure of complexity was selected as it can be employed with comparatively short time series and is robust regarding outliers (Krane-Gartiser et al. 2014). For the analysis, data were normalized by transforming the time series to have sample mean 0 and sample variance 1. Sample entropy is the negative natural logarithm of an estimate of the conditional probability that subseries of a certain length (*m*) that match point-wise, within a tolerance (*r*), also match at the next point (Hauge et al. 2011). We chose the following values, *m*  =  2 and *r*  =  0.2. A high value of sample entropy indicates a time series with high complexity or more disorder, while a low value indicates a more regular time series (Richman and Moorman 2000).
- Fourier analysis is an approach that is widely used in cardiology (to characterize heart rate variability and predictability); it explores (or decomposes) frequencies or periodic functions that form a harmonic series, thus allowing a complicated signal to be expressed in terms of the frequencies of the waves that make up the signal. It can be performed by taking a series of numbers along the time axis and the wave function (usually amplitude, frequency, or phase versus time) that can be expressed as a function called a Fourier series (uniquely defined by constants known as Fourier coefficients). In this study, data were normalized before analysis and results are presented as the relation between variance in the high-frequency part of the spectrum (0.0021–0.0083 Hz, corresponding to the period from 2 to 8 min) and the low-frequency part (0.00026–0.0021 Hz, corresponding to 8–64 min). A higher number indicates more variance in the high-frequency part as compared to the low-frequency parts of the spectrum.

### Statistics

All analyses were undertaken using SPSS (version 22), with statistical significance set at *p* < 0.5.

Age and activity parameters are described by mean values (and 95% CI) for each group (this approach was used to provide an indication of the upper and lower limit of the estimated mean). We undertook a preliminary analysis using MANOVA to determine if there were any overall differences in activity patterns according to group (defined by phase of illness), gender, and age (this analysis is not discussed in detail as some of the data are reported in other publications). Also, this study focuses on combinations of variables, so the findings of the MANOVA were used only to determine if age and gender should be included in the DFA.

Discriminant function analysis is a multiple regression technique that determines the best weighting of variables to maximize the differences among groups and predict group membership (Huberty and Olejnik 2006). We undertook a DFA with bootstrapping (1000 samples) and employed the standardized residual scores for each activity parameter as predictors in the model. We report Eigen values, explained variance, and Wilks’ Lambda (with Chi squared and statistical significance) for the canonical discriminant functions. We also show the structure matrix of correlations between discriminating variables and the functions (given the sample size, loadings of ≤.3 were considered uninterpretable; however, details are provided in the Additional file 1). Lastly, we report the proportion of cases correctly classified by the DFA, and note the reliability of this classification using a ‘leave-one-out’ cross-validation. In the cross-validation analysis, each case is deleted in turn, and the remaining observations are reclassified as per the rule established in the original model (Huberty and Olejnik 2006).

## Results

The sample mean age was 44.6 years (95% CI 38.56–50.62). As shown in Table 1, just over half of the sample was female (56%) and just under half was manic (47%). All patients were being prescribed psychotropic medications at the time of admission. Manic cases were non-significantly older than the other subgroups.

**Table 1:** Sample demographics

Gender	Phase of illness	Number (%)	Age in years		
			Mean	95% CI	
Males	Bipolar depression	5 (33%)	39.20	24.70	53.70
	Mania	7 (47%)	55.29	43.03	67.54
	Mixed state	3 (20%)	41.00	22.27	59.73
Females	Bipolar depression	7 (37%)	40.43	28.17	52.69
	Mania	9 (47%)	48.64	38.86	58.42
	Mixed state	3 (16%)	43.00	24.27	61.73
Total sample	Bipolar depression	12 (35%)	39.92	29.99	49.84
	Mania	16 (47%)	51.22	43.58	58.86
	Mixed state	6 (18%)	42.00	26.65	57.35

Table 2 provides details regarding the six activity parameters recorded in the active morning and evening periods for each phase of illness. The MANOVA showed that the overall pattern of activity differed significantly between groups (*F* = 2.81, *p* = 0.028), and also by age (*F* = 2.69, *p* = 0.037), but not by gender (*F* = 1.25, *p* = 0.39).

**Table 2:** Estimates of parameters for the 64-min active morning and evening periods according to phase of bipolar disorder, adjusted for multiple comparisons, and controlling for age and gender

Activity parameter	Phase of illness	Morning period			Evening period		
		Mean	95% CI		Mean	95% CI	
Mean activity count per minute	Bipolar depression	218.53	132.02	305.04	154.86	95.98	213.75
	Mania	229.80	154.24	305.37	239.34	187.91	290.78
	Mixed state	281.72	164.41	399.02	254.49	174.65	334.33
SD in % of mean activity count	Bipolar depression	117.42	98.88	135.96	129.48	104.58	154.37
	Mania	90.04	73.84	106.24	92.11	70.36	113.85
	Mixed state	85.68	60.54	110.83	111.13	77.38	144.88
RMSSD in % of mean activity	Bipolar depression	108.12	85.20	131.05	124.81	96.79	152.84
	Mania	88.75	68.72	108.78	90.75	66.26	115.23
	Mixed state	84.71	53.62	115.80	97.61	59.61	135.61
Autocorrelation	Bipolar depression	.56	.46	.67	.53	.43	.62
	Mania	.49	.40	.58	.49	.40	.58
	Mixed state	.51	.37	.65	.60	.47	.74
Sample entropy	Bipolar depression	1.05	.70	1.40	1.03	.65	1.41
	Mania	1.41	1.11	1.72	1.36	1.03	1.69
	Mixed state	1.55	1.08	2.03	.96	.44	1.47
Fourier analysis (2–8 min/8–64 min)	Bipolar depression	.67	.38	.96	.77	.41	1.14
	Mania	.90	.64	1.15	1.05	.73	1.37
	Mixed state	.97	.58	1.37	.58	.08	1.07

 Tables 3 and 4 shows that a DFA using the activity variables as predictors revealed two functions. The first function had an Eigen value of 3.14, was significant (*p* = 0.027), and explained 75% of the variance in the ‘boot-strapped’ model (Canonical correlation = .87) and maximally separated the BD groups. The second function in the model had a Canonical correlation of .71, but Wilks’ Lambda was non-significant (see Table 3).

**Table 3:** Discriminant analysis: Eigen values and Wilks’ Lambda for canonical discriminant functions

Function	Eigen values	% of variance	Canonical correlation	Wilks’ lambda	Chi square	Sig.
1	3.04	75	.87	.12	49.19	.027
2	1.01	25	.71	.49	16.36	.358

**Table 4:** Discriminant analysis: structure matrix showing pooled within-group correlations between discriminating variables and standardized canonical discriminant functions

Activity parameter	Function	
	1	2
SD in % of mean activity count (morning period)	−.41	
Sample entropy (morning period)	.35	
Fourier analysis (2–8 min/8–64 min of the evening period)		−.53
Autocorrelation (evening period)		.44
Sample entropy (evening period)		−.38
SD in % of mean activity count (evening period)		.36

Table 4 provides details of the structure matrix canonical loadings of the predictor variables and the two discriminant functions. It indicates that the first function was correlated with activity in the morning period, with measures of intra-individual fluctuation in activity (SD in % of mean activity count) and regularity of activity (sample entropy) showing the strongest associations. The second function correlated with several measures of activity from the evening period, namely the Fourier analysis, autocorrelation, sample entropy, and the SD in % of mean activity count.

**Fig. 1:**
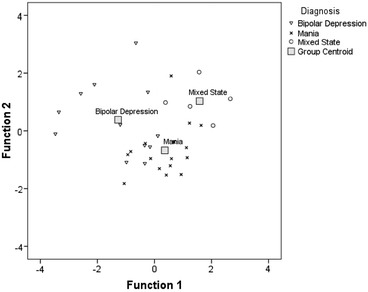
Canonical discriminant functions by diagnosis. *Note* Functions at group centroids: bipolar depression (function 1 = −1.28; function 2 = 0.38); mania (0.36; −0.68); and mixed states (1.58; 1.03)

 Figure 1 provides a graphic representation of the distribution of the cases, demonstrating that BDep cases are more widely dispersed (from their group centroid) than cases of mania or mixed states.

 As can be seen in Table 5, a classification table generated using both functions revealed that 79% of original BD cases were correctly categorized based on phase of illness. Although the overall model is statistically significant (*χ* ^2^ = 36.21; *df* 4; *p* = 0.001), it is noticeable that the classification rates are high for both mania and mixed states, but that 42% of BDep cases are misclassified as manic. The cross-validated classification results largely supported this model (details not shown), although it was less diagnostically accurate (55%) than the original classification; BDep misclassifications were still the most frequent, but there were a small number of misclassifications of mania cases as mixed episodes and vice versa. (A secondary analysis of BDep cases did not find any significant differences in classification accuracy for BD-I or -II; although subgroup samples were small).

**Table 5:** Proportion of cases correctly classified by discriminant function analysis

Original group	Predicted group			Proportion correctly classified
	Bipolar depression	Mania	Mixed state	
Bipolar Depression	7 (58%)	5 (42%)	0	79% (χ^2^ = 36.21; sig. = 0.001)
Mania	0	15 (94%)	1 (6%)	
Mixed state	0	1 (17%)	5 (83%)	

## Discussion

The discussion is organized into three broad topics. First, the interpretation of the findings in the context of diagnostic criteria for BD and research on activity in different illness phases; second, consideration of the limitations of the study; and third, the implications of the methodology for the role of actigraphy and BD.

Many researchers have welcomed the fact that the DSM-5 recognizes motor changes in relation to (hypo)manic episodes, but were disappointed that this diagnostic criterion was not extended to BDep or mixed states. Another notable omission from the DSM-5 criterion, was that no details were provided as to whether the abnormalities in activity refer to the mean level, the timing, or the overall pattern, etc. Scott et al. (2016a) suggest that a more nuanced description of activity is important in improving our understanding of the phenomenology of different phases of BD and further improving diagnostic criteria, and argue that subtle differences in activity are unlikely to be captured by the currently available symptom rating scales. As such, the availability of objective measures of activity over time may offer critical insights into the ‘non-mood’ differences between each phase of BD. Using this approach, our study produced three important findings. First, in acutely admitted BD cases, we identified that measures of variability and entropy of activity in the morning significantly differentiated BDep from mixed states or mania. Likewise, the second factor identified by DFA (although not statistically significant) comprised dynamic measures of evening activity, not mean levels. These findings confirm the importance of considering sets of activity parameters that extend beyond simple actigraphy measures (such as mean counts per minute) to include variability, regularity, amplitude, and 24-h patterning or predictability of rest-activity cycles. Second, a model incorporating both factors identified by the DFA successfully classified four out of five cases based on phase of illness; being particularly useful for mania and mixed states. Third, the model only improved the classification of BDep cases to 58% (12 of 34 cases were diagnosed as BDep at study inclusion giving a prior probability of 29%) and, in addition, the cross-validation was less accurate than the original classification model. One interpretation of these data is that they reflect weaknesses in the final model. Another is that the BDep group was more heterogenous than other subgroups. In the current sample, it is not possible to fully examine this possibility, although we note that BD subtype (I or II) did not predict accuracy of classification. The BDEp cases that were misclassified appeared to have activity profiles that were like manic cases. This is interesting in the context of research that argues against a unidimensional model of BD (that suggests that depressed and elated mood are polar opposites), and that argues for the notion that changes in mood states and activity may be independent of each other (DiSalver et al. 1999; Johnson et al. 2011). Whilst the current study is inconclusive, the methodology offers a potential strategy for future investigations of the temporal relationship between activity and mood, and may contribute to debates about whether there are different subtypes of BDep (Perich et al. 2016).

The study is the first to include a separate group with mixed episodes, and to apply DFA to activity parameters. However, it has several limitations. Whilst the overall sample size compares closely to the medians reported for other actigraphy studies [for reviews see: Geoffroy et al. (2015); Ng et al. (2015); DeCrescenzo et al. (2016); Scott et al. (2016a)], it limited the number of statistical analyses that could be undertaken, and all the subgroup analyses must be seen as preliminary. Also, the duration of actigraphy recording was only 24 h and we focused on data from two selected 64-min periods of activity. Researchers in BD who undertake actigraphy studies have not yet used a strategy that targets an active morning and evening period, so we cannot make any cross-study comparisons to determine if our findings are representative of other clinical samples. We controlled for age and gender in our analyses, but we did not take account of confounding that might arise due to other factors known to affect rest-activity patterns ranging from body mass index through to the use of medications. These are potentially important, as acute changes in medication regime, reduced levels of adherence, or different treatment protocols for different illness phases could affect activity in our sample. We undertook the monitoring early to try to reduce the impact of changes in treatment, (as these may take some time to be fully effective), etc., but fully accept that the naturalistic approach and lack of control on treatments, ward protocols (the inpatient unit may have structured activity programmes, etc.), can potentially bias the findings. Having said that, we note that findings are inconsistent regarding the effects of class and dose of psychotropic medications on actigraphy outputs (Salvatore et al. 2008; Banihashemi et al. 2016; Scott et al. 2016b). Lastly, some researchers may argue that machine learning rather than DFA should have been used to explore case classification. We accept that the former technique is growing in popularity and is increasingly being applied in psychiatry. However, on balance, we identified that DFA was likely to simultaneously identify groups of inpatients with similar activity patterns and maximize between-group differences (Huberty and Olejnik 2006); whilst approaches such as machine learning have a potential for overfitting the model and generating a stable predictive model, using the latter would have required a much larger sample (probably > 120 cases) (Orru et al. 2015).

In the introduction, we highlighted that most BD studies employing actigraphy have focused on basic analyses of sleep data, with fewer studies exploring activity. To maximize the utility of actigraphy, it is important to extend data collection and analysis to the 24-h sleep-wake cycle, to avoid simplistic assumptions about the nature of activity patterns, and to extend the modelling to include algorithms that describe more sophisticated approaches such as ‘shape-naïve’ (Gershon et al. 2016), non-parametric (Goncalves et al. 2015), or non-linear analyses (e.g. Hauge et al. 2011). This will allow the examination of the dynamic characteristics of activation change in mania, mixed states and BDep (Scott et al. 2016a). To date, even studies that examine activity in BD form a heterogeneous group, with the topic addressed by comparing mean, maximum, and peak levels of activity, as well as the variability (over 24 h or across consecutive days), predictability or complexity of actigraphy. It would be valuable to try to elaborate a consensus view on key measures that might be examined across studies, as has been instigated in neuropsychological assessment (Green et al. 2004). This would enable a more rapid development of our understanding of motor activity in BD, and ensure that researchers are encouraged to report sleep-wake cycle data rather than sleep data alone and to consider these activity data within the context of circadian rhythms, etc. (Scott et al. 2016a). Lastly, it would help the development of a mathematical understanding of the relationship between simple mean level, variability, periodic, and non-linear perspectives of activity, and whether combinations of these variables may better describe different presentations of BD, which are likely to be critically important to the evolution of personalized medicine in mood disorders.

## Additional file

**Additional file 1.** Supplementary Material for Discriminant Functional Analysis of actigraphy parameters: Details of standardized canonical discriminant function correlates and structure matrix for all activity parameters included in the analyses.

## Abbreviations

AM: ante-meridian (morning)
BD: bipolar disorders
BDep: bipolar depression
CI: confidence intervals
DFA: discriminant function analysis
DSM-5: diagnostic and statistical manual of mental disorders, 5th edition
ICD-10: international classification of diseases, 10th edition
MANOVA: multivariate analysis of variance
PM: post-meridian (afternoon)
RMSSD: root mean squares of successive differences
SD: standard deviation
Sig.: significance
SPSS: statistical package for the social sciences
UP: unipolar
