# Supplementary material for: A pilot study to determine whether combinations of objectively measured activity parameters can be used to differentiate between mixed states, mania, and bipolar depression
Source: Int J Bipolar Disord. 2017 Mar 1;5:5. doi: 10.1186/s40345-017-0076-6 (PMC5331021; doi:10.1186/s40345-017-0076-6)
Supplement: Supplementary file 1 — Additional file 1. Supplementary Material for Discriminant Functional Analysis of actigraphy parameters: Details of standardized canonical discriminant function correlates and structure matrix for all activity parameters included in the analyses. [file 40345_2017_76_MOESM1_ESM.docx]

**Supplementary Material for Discriminant Functional Analysis of actigraphy parameters (Scott et al):**

**Details of standardized canonical discriminant function correlates and structure matrix for all activity parameters included in the analyses**

| 1. **Standardized Canonical Discriminant**   **Function Coefficients** | | |
| --- | --- | --- |
|  | Function | |
|  | 1 | 2 |
| Sample entropy (64-min active morning period) | -.430 | -.161 |
| Fourier analysis (2-8 min/8-64 min) (64-minute active morning period) | 1.418 | .367 |
| Autocorrelation (64-minute active morning period) | 4.546 | 2.489 |
| Mean activity count per minute (64-minute active morning period) | -.693 | .715 |
| SD in % of mean activity count (64-minute active morning period) | -8.243 | -3.942 |
| RMSSD in % of mean activity (64-minute active morning period) | 8.040 | 4.787 |
| Sample entropy (64-minute active evening period) | -.176 | .042 |
| Fourier analysis (2-8 min/8-64 min) (64-minute active evening period) | -.248 | -1.200 |
| Autocorrelation (64-minute active evening period) | -1.191 | -1.900 |
| Mean activity count per minute (64-minute active evening period) | 1.278 | -.061 |
| SD in % of mean activity count (64-minute active evening period) | 2.294 | 3.922 |
| RMSSD in % of mean activity count (64-minute active evening period) | -1.482 | -3.790 |

| 1. **Structure Matrix** | | | |
| --- | --- | --- | --- |
|  | Function | | |
|  | 1 | 2 | |
| SD in % of mean activity count (64-minute active morning period) | -.412^*^ | .294 | |
| Sample entropy (64-min active morning period) | .351^*^ | -.204 | |
| Mean activity count per minute (64-minute active evening period) | .294^*^ | .033 | |
| RMSSD in % of mean activity (64-minute active morning period) | -.248^*^ | .174 | |
| Fourier analysis (2-8 min/8-64 min) (64-minute active morning period) | .221^*^ | -.011 | |
| RMSSD in % of mean activity count (64-minute active evening period) | -.208^*^ | .140 | |
| Fourier analysis (2-8 min/8-64 min) (64-minute active evening period) | -.002 | -.532^*^ | |
| Autocorrelation (64-minute active evening period) | .063 | .448^*^ | |
| Sample entropy (64-minute active evening period) | .023 | -.383^*^ | |
| SD in % of mean activity count (64-minute active evening period) | -.214 | .362^*^ | |
| Mean activity count per minute (64-minute active morning period) | .087 | .273^*^ | |
| Autocorrelation (64-min active morning period) | -.135 | .178^*^ | |
| Pooled within-groups correlations between discriminating variables and standardized canonical discriminant functions | | |  |
| *Largest absolute correlation between each variable and any discriminant function | | |  |
